# Automated interpretation of influenza hemagglutination inhibition (HAI) assays: Is plate tilting necessary?

**DOI:** 10.1371/journal.pone.0179939

**Published:** 2017-06-29

**Authors:** Garrett Wilson, Zhiping Ye, Hang Xie, Steven Vahl, Erica Dawson, Kathy Rowlen

**Affiliations:** 1 InDevR Inc., Boulder, Colorado, United States of America; 2 Division of Viral Products, Office of Vaccines Research and Review, Center for Biologics Evaluation and Research, U.S. Food and Drug Administration, Silver Springs, Maryland, United States of America; University of Minnesota College of Veterinary Medicine, UNITED STATES

## Abstract

The hemagglutination inhibition assay (HAI) is widely used to evaluate vaccine-induced antibody responses as well as to antigenically characterize influenza viruses. The results of an HAI assay are based on an endpoint titration where the titers are generally manually interpreted and recorded by a well-trained expert. For serological applications, the lack of standardization in endpoint interpretation and interference from non-specific inhibitors in clinical samples can translate into a high degree of variability in the results. For example, tilting HAI plates at 45–60 degrees to look for a “tear drop pattern” with avian red blood cells is a common practice by many, but not all, research laboratories. In this work, we tested the hypothesis that an automated image analysis algorithm can be used to achieve an accurate and non-subjective interpretation of HAI assays—specifically without the need to tilt plates. In a side-by-side comparison study performed during FDA’s biannual serological screening process for influenza viruses, titer calls for more than 2200 serum samples were made by the Cypher One automated hemagglutination analyzer *without tilting* and by an expert human *with tilting*. The comparison yielded 95.6% agreement between the expert reader and automated interpretation method (within ± 1 dilution) for the complete dataset. Performance was also evaluated relative to the type of red blood cell (turkey and guinea pig) and influenza strain (12 different viruses). For the subset that utilized guinea pig red blood cells (~44% of the samples), for which no plate tilting was required, the agreement with an expert reader was 97.2%. For the subset that utilized turkey red blood cells (~56% of the samples), for which plate tilting was necessary by the expert reader, the agreement was 94.3%. Overall these results support the postulate that algorithm-based interpretation of a digital record with no plate tilting could replace manual reading for greater consistency in HAI assays.

## Introduction

The hemagglutination inhibition (HAI) assay is a conventional method utilized in various aspects of global influenza surveillance, diagnosis, antigenic characterization, and vaccine assessment. The HAI assay is required by the World Health Organization and other health authorities in the evaluation of annual influenza vaccine immunogenicity and cross-reactivity [1]. In addition to its criticality in influenza vaccine development and manufacturing, the HAI assay is also widely utilized within the veterinary and food safety industries to detect and characterize a range of microbial targets [2, 3]. Although the assay is relatively inexpensive and simple to perform, the lack of standardized reagents and consistent techniques have resulted in high variability between laboratories [4, 5].

For influenza viruses, hemagglutination is the process whereby the viral surface protein hemagglutinin binds to sialic acid sites on the surface of red blood cells (RBCs) thereby creating a sustained suspension of RBCs in solution. Typically RBCs of avian origin (chicken or turkey) or mammalian origin (horse or guinea pig) are used for analysis of influenza viruses. Hemagglutination inhibition titers are determined from the endpoint titration of antibodies in serum that bind to the virus, thereby inhibiting hemagglutination. HAI assays are conducted in 96-well U or V bottom plates, with each row or column consisting of a serial dilution of serum from a single sample. A fixed concentration of influenza virus antigen (4 HA units/25 μl) is added in each well to allow binding between hemagglutinin and anti-HA antibodies. Following an incubation period, a fixed concentration of RBCs is added to each well. If a sufficient number of anti-HA antibodies are present, hemagglutination will be inhibited or blocked and the RBCs will “precipitate” resulting in a solid red “button” (for avian-origin RBCs) or a hollow red “ring” (for mammalian-origin RBCs) at the bottom of the well that represents the non-agglutinated state. As the antibody concentration is decreased, hemagglutination will result and is visually observed as an opaque reddish haze within the well. The transition or endpoint between the non-agglutinated state and the agglutinated state is the titer (1/dilution factor).

In general, HAI assays are manually interpreted by visual examination of the endpoint within the dilution series. The consistency of human interpretation of HAI assay is challenged by the inherent subjectivity between different readers of various experience levels as well as the unpredictable presence of non-specific inhibition [4, 5, 6]. Non-specific inhibition may occur when endogenous proteins and/or inhibitors within the serum interfere with antibody-antigen interactions resulting in partial hemagglutination or irregular shapes of RBC precipitation, which is quite often seen with avian origin RBCs.

To aid in the sample interpretation when non-specific inhibition is present, many experienced readers will tilt the plate at a 45 to 65 degree angle for 30–60 seconds to look for a “tear drop” formed by avian RBCs. Samples which are considered truly non-agglutinated will have a clear “tear drop” pattern similar to the negative control, whereas samples that exhibit non-specific inhibition will not “fall” (or “stream”) at the same rate and/or with the same shape as the negative control [6]. If non-specific inhibition is detected, it is considered to be HAI negative (no antibodies present) and is assigned a titer value of less than the starting dilution.

A few automated instruments for analysis of hemagglutination reactions have been developed. Notable benefits of these instruments include increased standardization of interpretation, improved consistency of interpretation, and increased data integrity through digital record keeping. FluHema (SciRobotics, Israel) and Sanofi Pasteur VaxDesign (Florida USA) have both developed hemagglutination readers that automatically tilt and image the plate and assign the agglutination transition point [7]. InDevR’s Cypher One Automated Hemagglutination Analyzer (InDevR, Colorado USA) is the subject of the work described herein, and is an instrument that images plates without tilting. The system automatically assigns the agglutination transition point using a proprietary mathematical algorithm specifically developed to avoid the need to tilt the plate.

This report investigates the ability of the Cypher One system to accurately interpret HAI titers without tilting, for both avian and mammalian blood types, relative to the interpretation of an influenza expert with more than 25 years’ experience interpreting HAI assays.

## Materials and methods

### Sample preparation and HAI assay

Human serum samples (n = 2200) were de-identified, and data were analyzed anonymously by HAI assays against twelve different influenza viral antigens in the presence of either turkey or guinea pig RBCs. IRB approval was not sought for the data analysis included herein, as the de-identified samples utilized were part of the FDA's existing biannual serology testing. Serum samples underwent a standard receptor destroying enzyme (RDE) treatment at 37°C overnight followed by inactivation at 56°C for 30 min [6, 8, 9]. Phosphate buffered saline (PBS) was added to 96-well U-bottom plates (Costar 3797) at 25 μL/well for column 2–11 and 50 μL/well for column 12. Individual RDE-treated serum samples at initial 1:10 dilution were dispensed into each well of column 1 (i.e. A1, B1, C1, etc.) at 50 μL/well and were then serially (2-fold) diluted across the plate (i.e. A1, A2, A3, etc.) through Column 11. The excess 25 μL in all wells of column 11 was discarded. Pre-titrated antigens (8 HA units/50 μL) were then added to each plate at 25 μL/well for column 1–11. Column 12 was used as non-agglutinated negative controls containing neither serum nor virus. The plate was incubated at room temperature for 30 minutes to allow antibody-virus neutralization. After incubation, either 0.5% turkey RBCs or 0.75% guinea pig RBCs were added to each well at 50 μL/well for column 1–12 based on the antigens being tested [10]. The plate was again incubated at room temperature for 60 minutes.

### Human interpretation

For all plates within the turkey RBC dataset, the plate was first imaged in the Cypher One system (~40 seconds) and then immediately transferred to the experienced human reader for analysis. The time difference between the interpretation by the Cypher One system and the manual analysis by the human reader for all subsets was 20 minutes or less and still within the desired time window for accurate interpretation. For all experiments involving turkey RBCs, the plates, typically in a batch of four, were tilted at a 45 degree angle for 30–60 seconds allowing the RBCs in solution to migrate or stream to the edge of the well. In this time, the experienced human reader observed the motion of the RBC buttons in the control wells (column 12). Once the majority of the control wells completed formation of the tear-drop shape, the plate was set in a flat orientation on another fixture for further visual inspection. This fixture consisted of a platform that supported the edges of the 96-well plate while leaving the area on the underside of the plate free and unobstructed. A fixed 45 degree mirror was beneath the plate, allowing the experienced human reader to view the mirrored image of the bottom of the wells. Upon visually comparing the “teardrop” formation of each well relative to the negative control, the state of agglutination for each well is manually recorded as agglutinated (+) or non-agglutinated (-). Wells which visually appear to have tear drop formation rate and or shape similar to the negative control are considered non-agglutinated, whereas wells which form incomplete tear drops are considered agglutinated.

For experiments involving guinea pig RBCs, the plates were not required to be tilted prior to analysis and were first manually interpreted followed by imaging on the Cypher One system. The state of agglutination for guinea pig RBCs was determined by visually comparing the button morphology and density between the negative control and the well within question. The results were manually recorded as described for the turkey RBCs.

For both datasets the final titer value, represented as the inverse of the dilution factor, is the last non-agglutinated dilution within the series. In cases where no inhibition was observed, an HAI titer of 5 was assigned by the expert reader.

## Results and discussion

### *Cypher One* analysis

**Fig 1:**
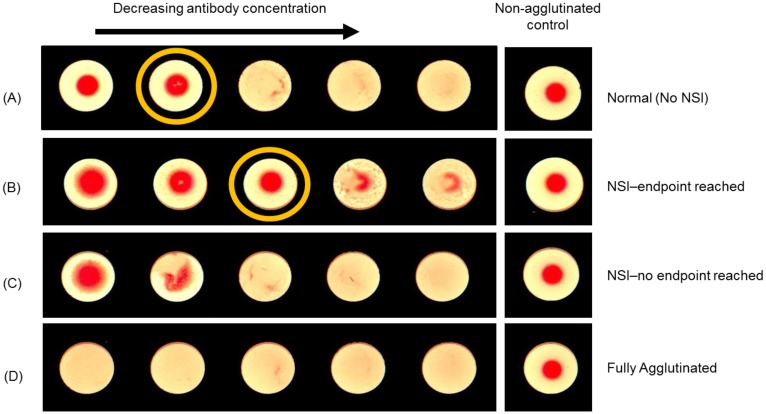
Morphological classifications within a sample titration. (A) HAI dilution series displaying a typical transition point in the absence of non-specific inhibition; a direct comparison of the first two wells to the negative control in the far right well shows very similar size, shape and overall morphology of the buttons. (B) HAI dilution series displaying non-uniform morphologies due to non-specific inhibition within the first well—an endpoint is reached in the third well; a direct comparison of the third well to the negative control in the far right well shows similar size, shape and overall morphology. (C) HAI dilution series displaying non-uniform morphologies due to non-specific inhibition; a direct comparison of the negative control in the far right well highlights the different morphology of the buttons as a function of dilution. (D) HAI dilution series displaying wells which are all fully agglutinated and have no inhibition as compared to the negative control in the far right well.

After incubation was complete, individual plates for each antigen subset were placed in the Cypher One instrument for imaging. The imaging event for each plate was completed within 40 seconds and a high resolution digital image, with roughly 2x the resolution achievable with the human eye, was displayed to the user. Fig 1 shows several examples of common turkey RBC “precipitate” morphologies observed in an HAI dilution series relative to a corresponding negative control. The yellow circle in row A and B indicate the selected endpoint or the last non-agglutinated well within the series. Fig 1A shows a normal HAI dilution series in the absence of any non-specific inhibition, which exhibits a clear transition from the non-agglutinated to agglutinated state. Fig 1B is an HAI dilution series where non-specific inhibition is present at low dilutions in the titration, but the series comes to a resolved endpoint at higher dilutions. In contrast, Fig 1C is an HAI dilution series in the presence of persistent non-specific inhibition that does not result in a non-agglutinated endpoint within the series. Fig 1D is an HAI serial dilution where all wells within the titration show agglutination. While these examples represent some of the more commonly observed morphologies, other variants exist that indicate the presence of non-specific inhibition, including poorly defined button edges, larger button diameters, and lower optical density compared to the negative control.

As a means to understand interpretation challenges caused by morphological differences, a careful manual examination of all images was performed in order to classify the image as one of the four distinct categories described in Fig 1. Specifically, the classifications are (i) normal (no NSI), (ii) NSI–endpoint reached, (iii) NSI–no endpoint reached, and (iv) fully agglutinated. Table 1 contains a summary of the number of samples assigned to each classification based on their morphological appearances. It is worth noting that the overall percentage of the classification ‘NSI–no endpoint reached’ was far greater in the turkey RBC dataset (24.2%) compared to the guinea pig RBC dataset (9.5%). This high incidence of non-specific inhibition for certain sample-RBC combinations illustrates why a tilting methodology is often employed.

**Table 1:** Sample classifications based on morphological appearances.

RBC Type	Classification	N	% of Dataset
**Turkey**	Normal (No NSI)	591	47.7%
	NSI—endpoint reached	236	19.1%
	NSI—no endpoint reached	299	24.2%
	Fully Agglutinated	112	9.0%
**Guinea Pig**	Normal (No NSI)	790	82.1%
	NSI—endpoint reached	33	3.4%
	NSI—no endpoint reached	91	9.5%
	Fully Agglutinated	48	5.0%

**Fig 2:**
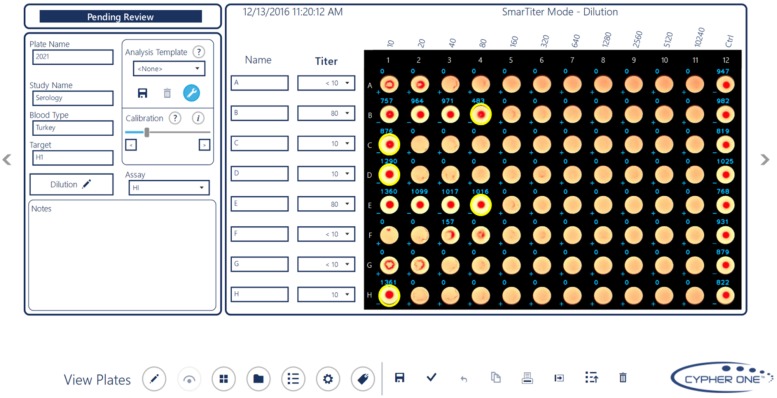
Screen shot of Cypher One user interface. Example HAI plate image within a screen shot of the Cypher One software application. High resolution digital images were captured and titer values determined using an image analysis algorithm within the software. The experimental information, image and resulting titer values are stored as digital records within the system database.

The Cypher One system uses a proprietary image analysis algorithm to evaluate the extent of agglutination in each well of a plate and the trend within each row (or column) to determine and display the titer value for each sample. The software is supplied with pre-defined settings for image analysis; however, the user can also define settings to match the titer calls of a specific human interpretation for a control plate, save those settings, and then apply those settings to an entire batch of plate images for automated analysis. This feature provides flexibility for labs with different historical titer call points (e.g., partial agglutination vs full agglutination). Experimental variables such as starting dilution, dilution factor, sample identification, plate name, blood type, virus type and notes were entered into the software to link the image and assay interpretation. A representative image from the user interface is shown in Fig 2.

The image analysis algorithm performs a mathematical comparison between the derived numerical outputs of sample wells and control wells to determine the state of agglutination of each well. There are two adjustable image analysis parameters within the algorithm. The first parameter (P1) may be roughly described as image contrast and defines the point where a pixel is considered high or low intensity. The output from this parameter is a unitless numerical value linked to each well. The second parameter (P2) is a “threshold” that is derived from the numerical value (output of P1) of the negative controls on the plate. The “threshold” (P2) defines the cutoff that delineates agglutination from non-agglutination. These two parameters can be optimized for a variety of sample types, including use of different RBC (e.g., guinea pig, turkey, equine) and RBC concentrations. Once the parameters have been optimized for a specific experimental configuration, they can be fixed for future experiments of the same type. In this study, both parameters were tuned using a systematic optimization routine to select a parameter combination to match the human reader’s tendencies for each antigen set. The optimized settings per antigen set were then conserved (fixed) and applied to all samples within that antigen set. Consistent with the titer call made by the experienced human reader, the final titer value, represented as the inverse of the dilution factor, is the last non-agglutinated dilution within the series.

### Comparison of results

For each sample, Cypher One titer values for both turkey RBCs and guinea pig RBCs were compared to the titer values determined by the experienced human reader. Since the hypothesis being tested was whether or not plate tilting is essential, the accuracy was purposely defined with respect to a single, highly experienced reader. This helped minimize variables caused by differences in experimental and/or interpretation techniques. Such an approach is reasonable given that the two image analysis parameters can be adjusted to meet the interpretation habits of a given laboratory or expert. Accuracy is further defined as the agreement within ± 1 dilution (generally a 2-fold difference) of the titer call made by the experienced reader. This 2-fold difference from a reference titer value is generally considered an equivalent result [3, 11, 12]. To challenge this benchmark, we conducted a survey to evaluate the self-consistency of the interpretation of several experienced readers using a large blinded dataset of images that included numerous replicates (i.e., users were asked to call the titer for the same sample shown to them randomly). Results indicated that human readers are only able to achieve ~95% agreement (± 1 dilution) *with their own interpretation* (data not shown), supporting the assertion that titer values that differ by ± 1 dilution from the human-interpreted reference titer can be considered a reasonable benchmark.

**Fig 3:**
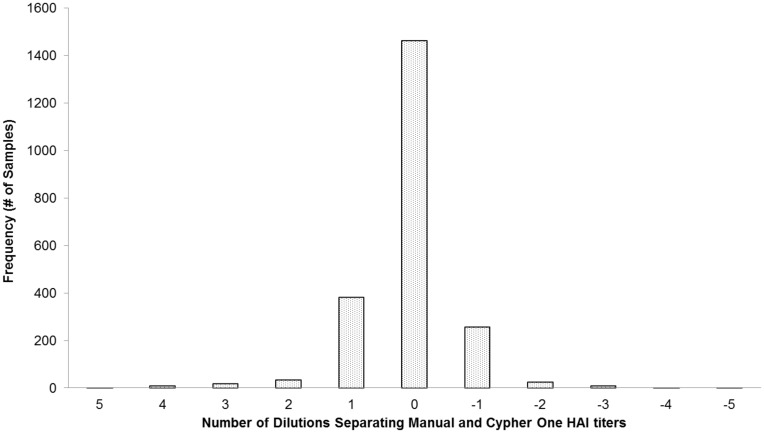
Histogram representing the number of dilutions different between the Cypher One and human titer interpretation for the entire dataset (n = 2200).

Since the sample set is large and includes a wide range of titer values, the data were evaluated as the absolute difference in number of dilutions (i.e. 1 dilution, 2 dilutions) between interpretation methods, with ± 1 dilution relative to expert reader’s titer call considered an equivalent result for calculation of the overall percent agreement. Fig 3 is a histogram of the “number of dilutions” (i.e., wells) that separated the expert reader and Cypher One HAI titer calls for the entire dataset of 2200 samples. Overall, 95.6% the titer calls made by Cypher One were within ± 1 dilution of the manual titer calls, indicating a high level of agreement to the experienced human reader.

Histogram showing the difference in Cypher One titer calls compared to the expert human reader in terms of the number of dilutions separating the two interpretations for the entire dataset.

To investigate performance in greater detail, Table 2 contains a summary of the percent agreement within ± 1 dilution for each blood type and for each of the antigen sets investigated in the serological screening study. For samples screened using guinea pig RBCs, no plate tilting was performed. Of the 962 samples tested within the guinea pig dataset, 935 (97.2%) had a Cypher One call within ±1 dilution of the expert interpretation. Thus, the Cypher One system exhibited a high degree of agreement with the expert reader. It is interesting to note that the morphology of the “precipitate” for guinea pig RBCs often represents an interpretation challenge for inexperienced human readers.

**Table 2:** Cypher One agreement to expert human reader.

RBC Type	Antigen	N	P1 (no units)	P2 (%)	% Agreement (+/- 1 Dilution)
**Turkey**	1 (H1N1 A/Michigan/45/2015) Egg-grown	193	95	70	99.5%
	2 (B/Florida/78/2015) Cell-grown	94	105	70	90.4%
	3 (B/Brisbane/60/2008) Egg-grown	193	85	60	94.3%
	4 (B/Florida/78/2015) Egg-grown	193	80	50	90.7%
	5 (B/Arizona/10/2015) Egg-grown	190	85	80	92.6%
	6 (H1N1 A/California/07/2009) Egg-grown	193	80	50	94.8%
	7 (B/Phuket/3073/2013) Egg-grown	182	80	50	96.2%
	**Combined Subset Total**	**1238**	**--**	**--**	**94.3%**
**Guinea Pig**	8 (H3N2 A/Alaska/232/2015) Egg-grown	190	170	60	98.4%
	9 (H3N2 A/Texas/88/2016) Cell-grown	193	170	60	95.9%
	10 (H3N2 A/Hong Kong/4801/2014) Cell-grown	193	170	50	95.9%
	11 (H3N2 A/Switzerland/9715293/2013) Egg-grown	193	180	60	94.8%
	12 (H3N2 A/Hong Kong/4801/2014) Egg-grown	193	185	70	96.4%
	**Combined Subset Total**	**962**	**--**	**--**	**97.2%**

All of the plates containing turkey RBCs were tilted and analyzed by the expert reader after imaging by the Cypher One system. Of the 1238 samples tested within the turkey RBC dataset, 1168 (94.3%) had a Cypher One call within ±1 dilution of the expert’s titer call. Only 70 samples within the turkey RBC dataset (5.7%) exhibited a Cypher One call that was greater than ±1 dilution outside of the call of the human reader. Forty-seven (47) of the 70 outlier samples had a Cypher One titer call *higher* than that of the human reader, and for these samples, it was generally observed that the human reader assigned a titer value of 5 indicating non-specific inhibition across the row. When the archived Cypher One digital images were examined visually, it was confirmed that generally this group (assigned titer of 5 by human reader) did exhibit signs of non-specific inhibition. This indicates that a small percentage of samples (3.8% of entire dataset) with non-specific inhibition do represent a challenge for the Cypher One algorithm. In 23 of the 70 outlier samples (1.9% of the entire dataset), the Cypher One system assigned a titer value of 5. Given that the experienced reader did not assign a value of 5, it is assumed that these samples did not exhibit non-specific inhibition and the reason for disagreement is not clear.

In order to better understand the observed differences for samples that resulted in poor agreement, all of the samples were assigned to one of the four classifications described in Fig 1. Table 3 contains a summary of the percent agreement within ± 1 dilution for each sample classification. Between the blood types, the turkey dataset had a large percentage (43.2%) of samples that were categorized as “challenging” and were classified as either the NSI–endpoint reached or NSI–no endpoint reached; whereas the guinea pig dataset had only 12.9% that were assigned those classifications. For challenging samples within the guinea pig dataset (no plate tilting) an overall agreement of 89.5% (within ± 1 dilution) between interpretation methods was achieved. For challenging samples within the turkey RBC dataset (with plate tilting) an overall agreement of 89.7% (within ± 1 dilution) between interpretation methods was achieved. It is important to note that the performance, even for these challenging samples, is essentially the same for plates that required the human reader to tilt (turkey RBCs) and those that didn’t (guinea pig RBCs). This observation supports the hypothesis that no plate tilting is required for accurate interpretation by the Cypher One system.

**Table 3:** Cypher One agreement to expert human reader per sample classification.

RBC Type	Classification	N	% Agreement (+/- 1 Dilution)
**Turkey**	Normal (No NSI)	591	97.5%
	NSI—endpoint reached	236	90.3%
	NSI—no endpoint reached	299	89.3%
	Fully Agglutinated	112	100.0%
**Guinea Pig**	Normal (No NSI)	790	98.2%
	NSI—endpoint reached	33	87.9%
	NSI—no endpoint reached	91	90.1%
	Fully Agglutinated	48	100.0%

It is worth noting in Table 2 that the two antigens with the lowest percent agreement (~90%) within the turkey RBC dataset were both B/Florida/78/2015 strains, one egg-grown and one cell-grown. Further analysis (data not shown) of the egg-grown B/Florida/78/2015 strain shows that this antigen produced the highest percentage (49.7%) of combined samples that were classified as NSI–no endpoint reached and NSI–endpoint reached. The B/Florida/78/2015 strain (cell-grown) also had a high percentage (47.9%) of both NSI–no endpoint reached and NSI-endpoint reached.

**Fig 4:**
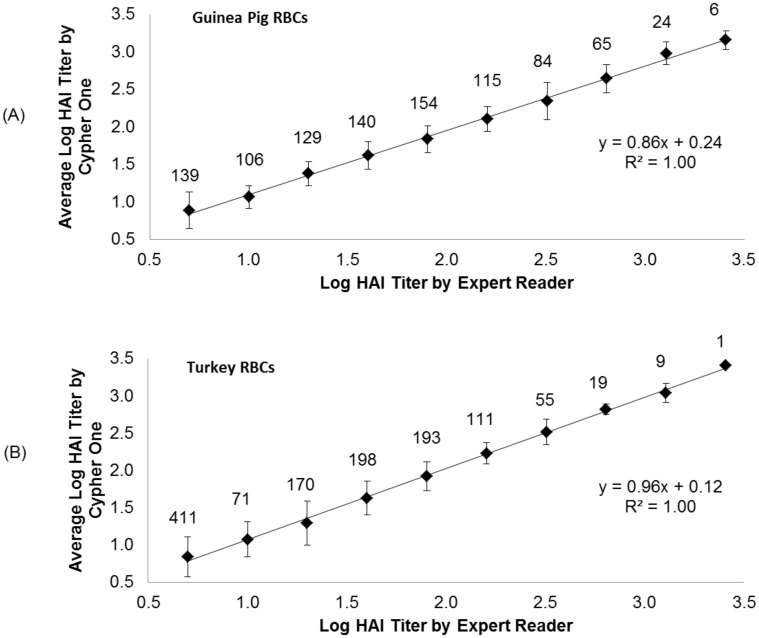
Log titer correlation plot for the guinea pig and turkey RBC datasets. (A) Shows the log titer correlation plot between interpretation methods for each assigned titer value for the guinea pig dataset. (B) Shows the log titer correlation plot between interpretation methods for each assigned titer value for the turkey dataset. The range of log titer values shown on the X axis reflect the titer values assigned by the human reader. The average Cypher One interpretation at all titer values is represented on the Y axis. Assigned titer values ranged from 5 to 2560, or log titer values of 0.7 to 3.4, respectively. Log titer values of 0.7 (HAI titer of 5) exhibited either non-specific inhibition or full agglutination across the entire dilution series. The number shown above each data point indicates the number of samples included in each data point.

Another metric for agreement between an expert reader and Cypher One system is the correlation in titer calls. Since the range of titer calls is large, log-log correlation plots for the guinea pig and turkey RBC datasets are shown in Fig 4. The number above each data point in the correlation plot is the total number of samples with a specific titer call as defined by the expert reader call. The error bars represent ± 1 standard deviation from the average Cypher One titer call at each of the titer values. For example, in the turkey RBC dataset 71 samples resulted in a log titer of 1.0 by the experienced human reader (HAI titer = 10). For those 71 samples, the average Cypher One titer value was calculated and plotted against the manually determined titer.

For the guinea pig data set the linear regression yielded a slope of 0.86 and a Pearson’s correlation coefficient of 1.0. A perfect linear correlation would result in a slope of 1.0 in a log-log plot; thus, a slope of 0.86 may imply a small systematic interpretation bias by either the expert reader or the algorithm. It is interesting to note that the guinea pig RBC dataset required a high “image contrast” setting (P1 value) to achieve optimized agreement with the expert reader. The log-log plot of titer calls for the turkey RBC dataset yielded a slope of 0.96 and a Pearson’s correlation coefficient of 1.0, indicating a strong linear correlation between the two interpretation methods.

## Conclusions

This study was conducted on a statistically significant dataset comprised of 2200 de-identified, archived serum samples and twelve different influenza antigens. Despite prevalent non-specific inhibition (17.7% of the entire data set), this study demonstrated a high (95.6%) overall agreement between the results from an expert human reader and those from the Cypher One system. Even in the presence of challenging samples where the endpoint was either non-existent or not obvious (>40% of the entire turkey RBC dataset), the Cypher One system did not require a tilting technique for accurate interpretation. Good agreement was also achieved the guinea pig RBC dataset which was interpreted by the expert human reader *without* tilting. Furthermore, the trend of agreement between the two interpretation methods is consistent and exhibits good correlation. Thus, we conclude that relative to manual interpretation by an experienced reader, Cypher One can provide a number of advantages. The Cypher One system eliminates the need to tilt prior to interpretation of images, simplifying the assay and reducing the time to result. Cypher One also generates a permanent digital record for each plate, with each image collected under the same imaging conditions for all plates. This imaging stability in combination with the use of a consistent mathematical algorithm for interpretation serves to minimize the both the subjectivity between different readers of various experience levels and possible data transcription errors. In addition, the entire data collection and analysis process can be logged with an audit trail tied to original results, thereby ensuring data integrity.

## Supporting information

S1 File (XLSX)
